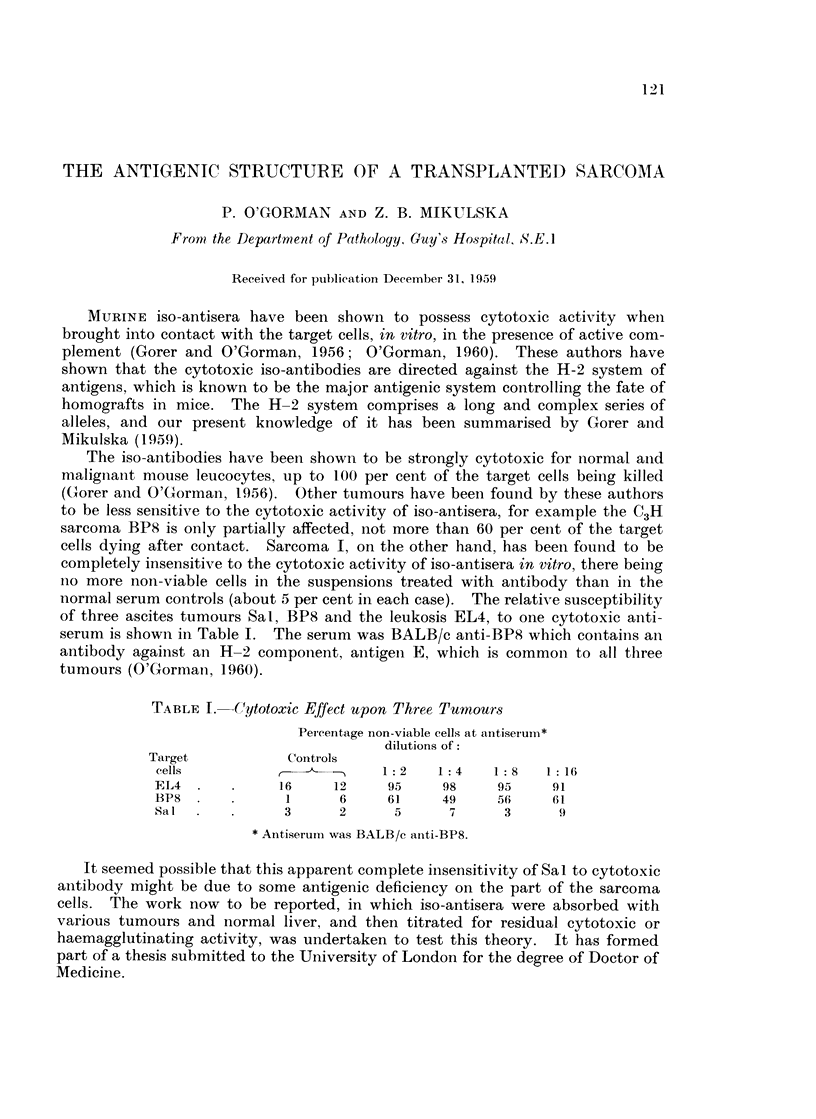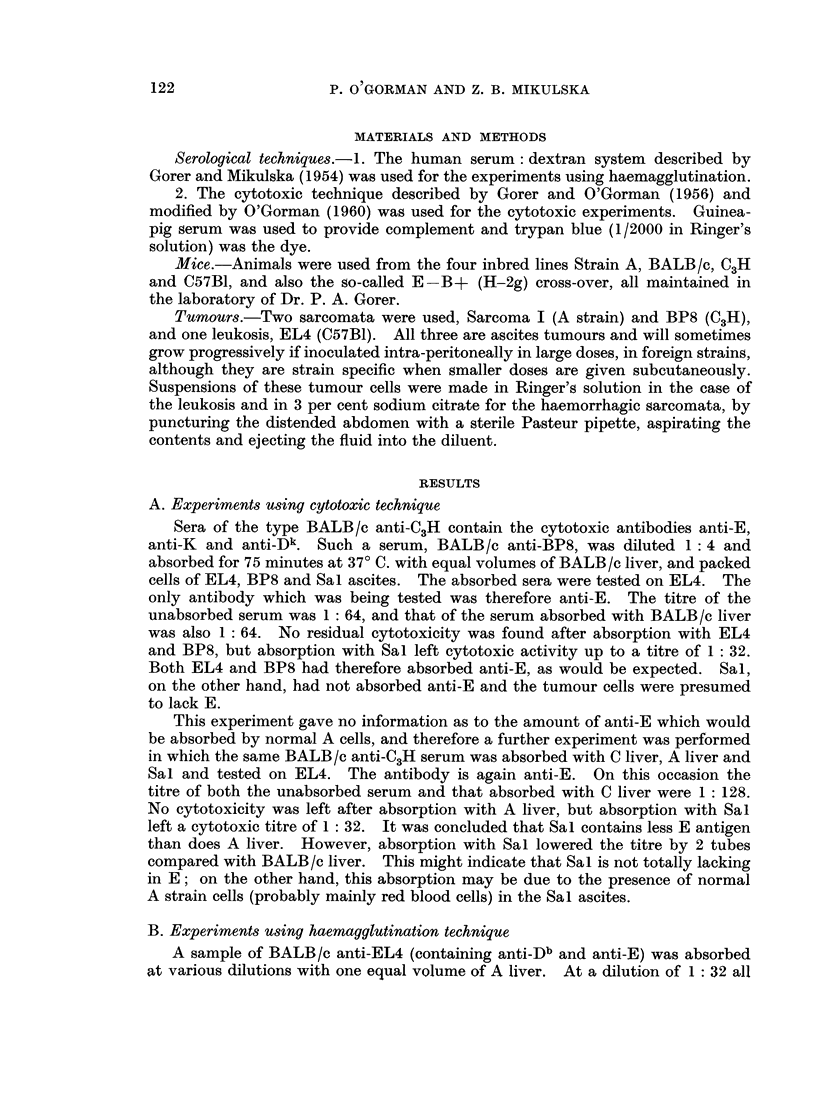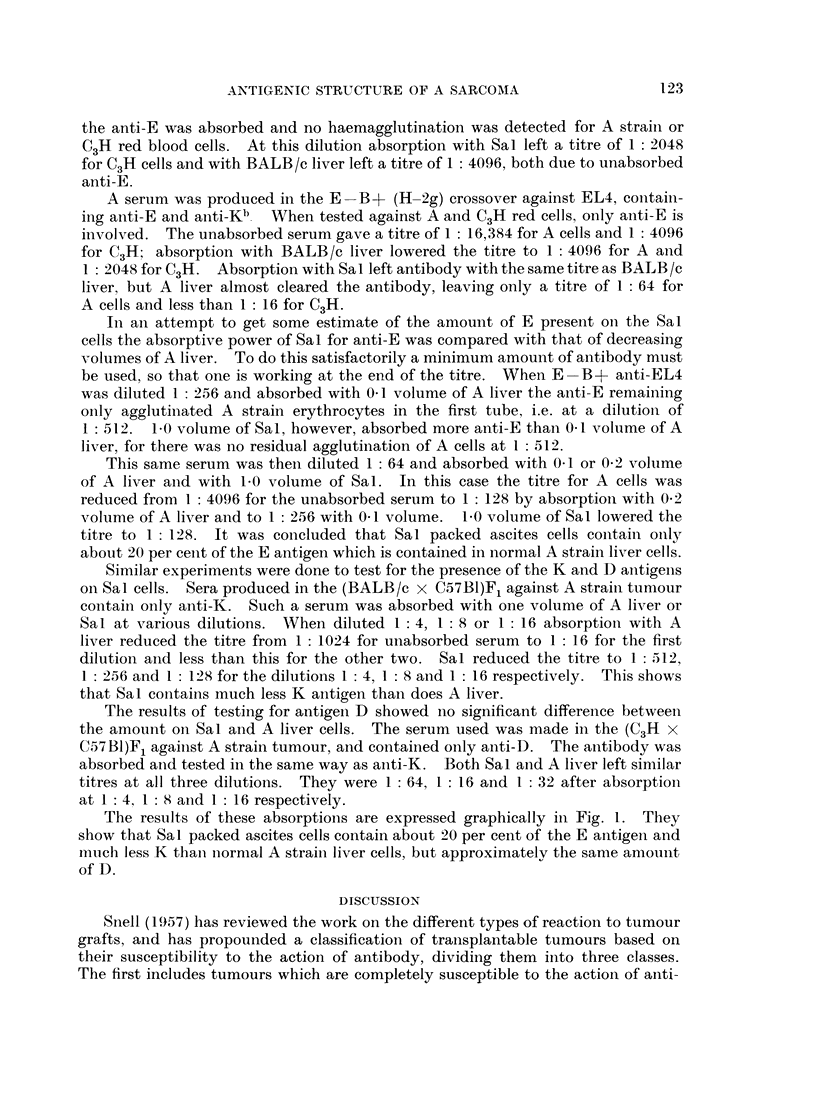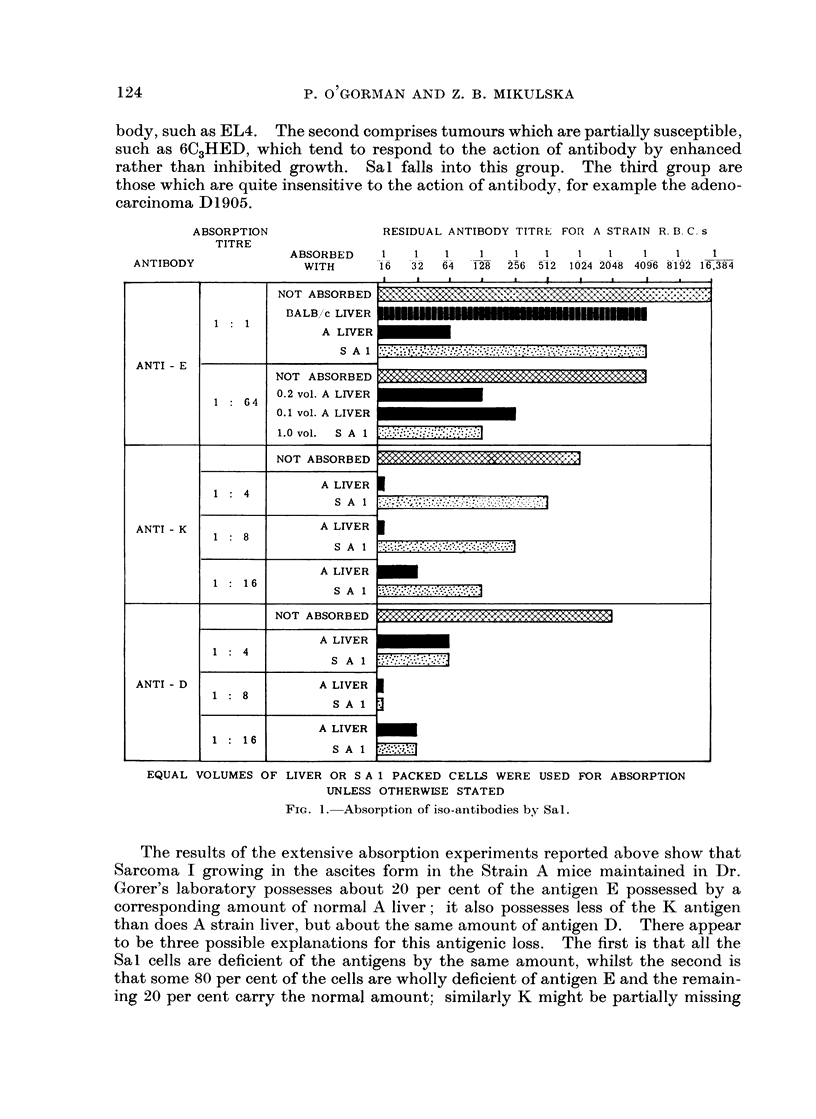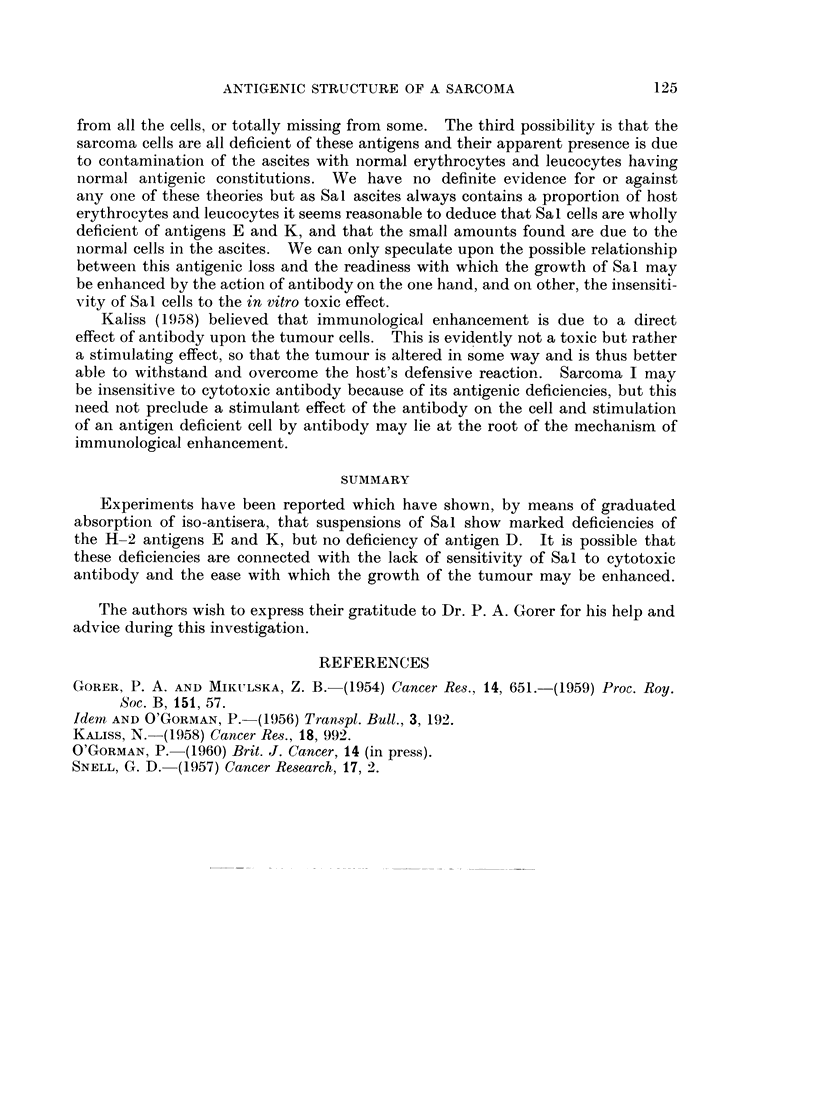# The Antigenic Structure of a Transplanted Sarcoma

**DOI:** 10.1038/bjc.1960.14

**Published:** 1960-03

**Authors:** P. O'Gorman, Z. B. Mikulska


### THE ANTIGENIC STRUCTURE OF A TRANSPLANTED SARCOMA

P. O'GORMAN AND Z. B. MIKULSKA

*From the Department of Pathology, Guy's Hospital, S.E.1*

Received for publication December 31, 1959

MURINE iso-antisera have been shown to possess cytotoxic activity when brought into contact with the target cells, *in vitro*, in the presence of active complement (Gorer and O'Gorman, 1956; O'Gorman, 1960). These authors have shown that the cytotoxic iso-antibodies are directed against the H-2 system of antigens, which is known to be the major antigenic system controlling the fate of homografts in mice. The H–2 system comprises a long and complex series of alleles, and our present knowledge of it has been summarised by Gorer and Mikulska (1959).

The iso-antibodies have been shown to be strongly cytotoxic for normal and malignant mouse leucocytes, up to 100 per cent of the target cells being killed (Gorer and O'Gorman, 1956). Other tumours have been found by these authors to be less sensitive to the cytotoxic activity of iso-antisera, for example the $C_3H$ sarcoma BP8 is only partially affected, not more than 60 per cent of the target cells dying after contact. Sarcoma I, on the other hand, has been found to be completely insensitive to the cytotoxic activity of iso-antisera *in vitro*, there being no more non-viable cells in the suspensions treated with antibody than in the normal serum controls (about 5 per cent in each case). The relative susceptibility of three ascites tumours Sa1, BP8 and the leukosis EL4, to one cytotoxic antiserum is shown in Table I. The serum was BALB/c anti-BP8 which contains an antibody against an H–2 component, antigen E, which is common to all three tumours (O'Gorman, 1960).

TABLE I.—*Cytotoxic Effect upon Three Tumours*

| Target cells | Controls | | Percentage non-viable cells at antiserum* dilutions of: 1 : 2 | 1 : 4 | 1 : 8 | 1 : 16 |
|---|---|---|---|---|---|---|
| EL4 | 16 | 12 | 95 | 98 | 95 | 91 |
| BP8 | 1 | 6 | 61 | 49 | 56 | 61 |
| Sa1 | 3 | 2 | 5 | 7 | 3 | 9 |

* Antiserum was BALB/c anti-BP8.

It seemed possible that this apparent complete insensitivity of Sa1 to cytotoxic antibody might be due to some antigenic deficiency on the part of the sarcoma cells. The work now to be reported, in which iso-antisera were absorbed with various tumours and normal liver, and then titrated for residual cytotoxic or haemagglutinating activity, was undertaken to test this theory. It has formed part of a thesis submitted to the University of London for the degree of Doctor of Medicine.

### MATERIALS AND METHODS

*Serological techniques.*—1. The human serum : dextran system described by Gorer and Mikulska (1954) was used for the experiments using haemagglutination.

2. The cytotoxic technique described by Gorer and O'Gorman (1956) and modified by O'Gorman (1960) was used for the cytotoxic experiments. Guinea-pig serum was used to provide complement and trypan blue (1/2000 in Ringer's solution) was the dye.

*Mice.*—Animals were used from the four inbred lines Strain A, BALB/c, $C_3H$ and C57Bl, and also the so-called E−B+ (H–2g) cross-over, all maintained in the laboratory of Dr. P. A. Gorer.

*Tumours.*—Two sarcomata were used, Sarcoma I (A strain) and BP8 ($C_3H$), and one leukosis, EL4 (C57Bl). All three are ascites tumours and will sometimes grow progressively if inoculated intra-peritoneally in large doses, in foreign strains, although they are strain specific when smaller doses are given subcutaneously. Suspensions of these tumour cells were made in Ringer's solution in the case of the leukosis and in 3 per cent sodium citrate for the haemorrhagic sarcomata, by puncturing the distended abdomen with a sterile Pasteur pipette, aspirating the contents and ejecting the fluid into the diluent.

### RESULTS

### A. *Experiments using cytotoxic technique*

Sera of the type BALB/c anti-$C_3H$ contain the cytotoxic antibodies anti-E, anti-K and anti-$D^k$. Such a serum, BALB/c anti-BP8, was diluted 1 : 4 and absorbed for 75 minutes at 37° C. with equal volumes of BALB/c liver, and packed cells of EL4, BP8 and Sa1 ascites. The absorbed sera were tested on EL4. The only antibody which was being tested was therefore anti-E. The titre of the unabsorbed serum was 1 : 64, and that of the serum absorbed with BALB/c liver was also 1 : 64. No residual cytotoxicity was found after absorption with EL4 and BP8, but absorption with Sa1 left cytotoxic activity up to a titre of 1 : 32. Both EL4 and BP8 had therefore absorbed anti-E, as would be expected. Sa1, on the other hand, had not absorbed anti-E and the tumour cells were presumed to lack E.

This experiment gave no information as to the amount of anti-E which would be absorbed by normal A cells, and therefore a further experiment was performed in which the same BALB/c anti-$C_3H$ serum was absorbed with C liver, A liver and Sa1 and tested on EL4. The antibody is again anti-E. On this occasion the titre of both the unabsorbed serum and that absorbed with C liver were 1 : 128. No cytotoxicity was left after absorption with A liver, but absorption with Sa1 left a cytotoxic titre of 1 : 32. It was concluded that Sa1 contains less E antigen than does A liver. However, absorption with Sa1 lowered the titre by 2 tubes compared with BALB/c liver. This might indicate that Sa1 is not totally lacking in E; on the other hand, this absorption may be due to the presence of normal A strain cells (probably mainly red blood cells) in the Sa1 ascites.

### B. *Experiments using haemagglutination technique*

A sample of BALB/c anti-EL4 (containing anti-$D^b$ and anti-E) was absorbed at various dilutions with one equal volume of A liver. At a dilution of 1 : 32 all the anti-E was absorbed and no haemagglutination was detected for A strain or $C_3H$ red blood cells. At this dilution absorption with Sa1 left a titre of 1 : 2048 for $C_3H$ cells and with BALB/c liver left a titre of 1 : 4096, both due to unabsorbed anti-E.

A serum was produced in the E−B+ (H–2g) crossover against EL4, containing anti-E and anti-$K^b$. When tested against A and $C_3H$ red cells, only anti-E is involved. The unabsorbed serum gave a titre of 1 : 16,384 for A cells and 1 : 4096 for $C_3H$; absorption with BALB/c liver lowered the titre to 1 : 4096 for A and 1 : 2048 for $C_3H$. Absorption with Sa1 left antibody with the same titre as BALB/c liver, but A liver almost cleared the antibody, leaving only a titre of 1 : 64 for A cells and less than 1 : 16 for $C_3H$.

In an attempt to get some estimate of the amount of E present on the Sa1 cells the absorptive power of Sa1 for anti-E was compared with that of decreasing volumes of A liver. To do this satisfactorily a minimum amount of antibody must be used, so that one is working at the end of the titre. When E−B+ anti-EL4 was diluted 1 : 256 and absorbed with 0·1 volume of A liver the anti-E remaining only agglutinated A strain erythrocytes in the first tube, i.e. at a dilution of 1 : 512. 1·0 volume of Sa1, however, absorbed more anti-E than 0·1 volume of A liver, for there was no residual agglutination of A cells at 1 : 512.

This same serum was then diluted 1 : 64 and absorbed with 0·1 or 0·2 volume of A liver and with 1·0 volume of Sa1. In this case the titre for A cells was reduced from 1 : 4096 for the unabsorbed serum to 1 : 128 by absorption with 0·2 volume of A liver and to 1 : 256 with 0·1 volume. 1·0 volume of Sa1 lowered the titre to 1 : 128. It was concluded that Sa1 packed ascites cells contain only about 20 per cent of the E antigen which is contained in normal A strain liver cells.

Similar experiments were done to test for the presence of the K and D antigens on Sa1 cells. Sera produced in the (BALB/c × C57Bl)$F_1$ against A strain tumour contain only anti-K. Such a serum was absorbed with one volume of A liver or Sa1 at various dilutions. When diluted 1 : 4, 1 : 8 or 1 : 16 absorption with A liver reduced the titre from 1 : 1024 for unabsorbed serum to 1 : 16 for the first dilution and less than this for the other two. Sa1 reduced the titre to 1 : 512, 1 : 256 and 1 : 128 for the dilutions 1 : 4, 1 : 8 and 1 : 16 respectively. This shows that Sa1 contains much less K antigen than does A liver.

The results of testing for antigen D showed no significant difference between the amount on Sa1 and A liver cells. The serum used was made in the ($C_3H$ × C57Bl)$F_1$ against A strain tumour, and contained only anti-D. The antibody was absorbed and tested in the same way as anti-K. Both Sa1 and A liver left similar titres at all three dilutions. They were 1 : 64, 1 : 16 and 1 : 32 after absorption at 1 : 4, 1 : 8 and 1 : 16 respectively.

The results of these absorptions are expressed graphically in Fig. 1. They show that Sa1 packed ascites cells contain about 20 per cent of the E antigen and much less K than normal A strain liver cells, but approximately the same amount of D.

### DISCUSSION

Snell (1957) has reviewed the work on the different types of reaction to tumour grafts, and has propounded a classification of transplantable tumours based on their susceptibility to the action of antibody, dividing them into three classes. The first includes tumours which are completely susceptible to the action of antibody, such as EL4. The second comprises tumours which are partially susceptible, such as $6C_3HED$, which tend to respond to the action of antibody by enhanced rather than inhibited growth. Sa1 falls into this group. The third group are those which are quite insensitive to the action of antibody, for example the adenocarcinoma D1905.

EQUAL VOLUMES OF LIVER OR S A 1 PACKED CELLS WERE USED FOR ABSORPTION UNLESS OTHERWISE STATED

FIG. 1.—Absorption of iso-antibodies by Sa1.

The results of the extensive absorption experiments reported above show that Sarcoma I growing in the ascites form in the Strain A mice maintained in Dr. Gorer's laboratory possesses about 20 per cent of the antigen E possessed by a corresponding amount of normal A liver; it also possesses less of the K antigen than does A strain liver, but about the same amount of antigen D. There appear to be three possible explanations for this antigenic loss. The first is that all the Sa1 cells are deficient of the antigens by the same amount, whilst the second is that some 80 per cent of the cells are wholly deficient of antigen E and the remaining 20 per cent carry the normal amount; similarly K might be partially missing from all the cells, or totally missing from some. The third possibility is that the sarcoma cells are all deficient of these antigens and their apparent presence is due to contamination of the ascites with normal erythrocytes and leucocytes having normal antigenic constitutions. We have no definite evidence for or against any one of these theories but as Sa1 ascites always contains a proportion of host erythrocytes and leucocytes it seems reasonable to deduce that Sa1 cells are wholly deficient of antigens E and K, and that the small amounts found are due to the normal cells in the ascites. We can only speculate upon the possible relationship between this antigenic loss and the readiness with which the growth of Sa1 may be enhanced by the action of antibody on the one hand, and on other, the insensitivity of Sa1 cells to the *in vitro* toxic effect.

Kaliss (1958) believed that immunological enhancement is due to a direct effect of antibody upon the tumour cells. This is evidently not a toxic but rather a stimulating effect, so that the tumour is altered in some way and is thus better able to withstand and overcome the host's defensive reaction. Sarcoma I may be insensitive to cytotoxic antibody because of its antigenic deficiencies, but this need not preclude a stimulant effect of the antibody on the cell and stimulation of an antigen deficient cell by antibody may lie at the root of the mechanism of immunological enhancement.

### SUMMARY

Experiments have been reported which have shown, by means of graduated absorption of iso-antisera, that suspensions of Sa1 show marked deficiencies of the H–2 antigens E and K, but no deficiency of antigen D. It is possible that these deficiencies are connected with the lack of sensitivity of Sa1 to cytotoxic antibody and the ease with which the growth of the tumour may be enhanced.

The authors wish to express their gratitude to Dr. P. A. Gorer for his help and advice during this investigation.

### REFERENCES

GORER, P. A. AND MIKULSKA, Z. B.—(1954) *Cancer Res.*, **14**, 651.—(1959) *Proc. Roy. Soc.* B, **151**, 57.

*Idem* AND O'GORMAN, P.—(1956) *Transpl. Bull.*, **3**, 192.

KALISS, N.—(1958) *Cancer Res.*, **18**, 992.

O'GORMAN, P.—(1960) *Brit. J. Cancer*, **14** (in press).

SNELL, G. D.—(1957) *Cancer Research*, **17**, 2.